# Gastrointestinal Illness among Triathletes Swimming in Non-Polluted versus Polluted Seawater Affected by Heavy Rainfall, Denmark, 2010-2011

**DOI:** 10.1371/journal.pone.0078371

**Published:** 2013-11-07

**Authors:** Nina Majlund Harder-Lauridsen, Katrin Gaardbo Kuhn, Anders Christian Erichsen, Kåre Mølbak, Steen Ethelberg

**Affiliations:** 1 Department of Infectious Disease Epidemiology, Statens Serum Institut, Copenhagen, Denmark; 2 DHI Group, Hørsholm, Denmark; Amphia Ziekenhuis, Netherlands

## Abstract

Recent years have seen an increase in the frequency of extreme rainfall and subsequent flooding across the world. Climate change models predict that such flooding will become more common, triggering sewer overflows, potentially with increased risks to human health. In August 2010, a triathlon sports competition was held in Copenhagen, Denmark, shortly after an extreme rainfall. The authors took advantage of this event to investigate disease risks in two comparable cohorts of physically fit, long distance swimmers competing in the sea next to a large urban area. An established model of bacterial concentration in the water was used to examine the level of pollution in a spatio-temporal manner. Symptoms and exposures among athletes were examined with a questionnaire using a retrospective cohort design and the questionnaire investigation was repeated after a triathlon competition held in non-polluted seawater in 2011. Diagnostic information was collected from microbiological laboratories. The results showed that the 3.8 kilometer open water swimming competition coincided with the peak of post-flooding bacterial contamination in 2010, with average concentrations of 1.5x10^4^ *E. coli* per 100 ml water. The attack rate of disease among 838 swimmers in 2010 was 42% compared to 8% among 931 swimmers in the 2011 competition (relative risk (RR) 5.0; 95% CI: 4.0-6.39). In 2010, illness was associated with having unintentionally swallowed contaminated water (RR 2.5; 95% CI: 1.8-3.4); and the risk increased with the number of mouthfuls of water swallowed. Confirmed aetiologies of infection included *Campylobacter, Giardia lamblia* and diarrhoeagenic *E. coli*. The study demonstrated a considerable risk of illness from water intake when swimming in contaminated seawater in 2010, and a small but measureable risk from non-polluted water in 2011. This suggests a significant risk of disease in people ingesting small amounts of flood water following extreme rainfall in urban areas.

## Introduction

During the past 50 years, there has been a statistically significant increasing trend in the number of heavy rainfall events, and climate change models predict that this trend will continue, with a higher frequency of heavy rainfall episodes during the 21^st^ century, particularly in higher latitudes and tropical regions [1]. In affected areas heavy rainfall often results in flash flooding with a high risk of sewer overflows and potential microbial contamination of surface water posing a range of risks to human health [2-4]. The link between rainfall or heavy precipitation and outbreaks of infectious diseases such as campylobacteriosis and cryptosporidiosis has been demonstrated on several occasions [5-7], primarily through statistical correlations between rainfall patterns and historical disease outbreaks. The majority of the disease outbreaks examined were caused by ingestion of contaminated drinking water from public or private water supplies, but at present there is limited evidence available to determine the risk of disease for people who are in physical contact with contaminated flood water without ingesting large amounts of it. 

In most developed countries with safe supplies of drinking water, the possibility of contaminated drinking water following a flooding episode is relatively small, however sewage-contaminated flood water in gardens, streets, recreational areas and in the sea is considerably higher. Sewage-contaminated seawater has been shown to contain a range of pathogens, and exposed individuals may experience various disease symptoms such as skin rashes, conjunctivitis, sinus infections and in particular gastroenteritis [8,9]. Several studies have demonstrated an increased risk of illness associated with swimming in contaminated recreational waters as well as a relationship between reported symptoms and increased findings of faecal indicator bacteria or pathogens in the water [10-12], highlighting the importance of quantifying the risk associated with exposure to contaminated recreational water. However cohort studies are difficult to perform, and there are many challenges to conducting experiments involving recreational or professional swimming in documented microbiologically contaminated waters. 

On the 14^th^ August 2010 an unusually heavy rainfall in Denmark led to severe flooding in and around the Danish capital of Copenhagen causing sewers to overflow. The following morning Copenhagen hosted an international ironman triathlon competition consisting of a 3.8 km ocean water swim course, followed by a 180 km biking race, and finally a 42.2 km marathon. In the week following the competition independent reports indicated that a number of athletes had suffered from diarrhoea and abdominal pains after the competition. This prompted the authors to initiate a study with the aim of determining the extent and identifying the source of illness among the participating athletes. In this paper we describe the results of the study, including the symptoms arising after swimming in polluted and non-polluted seawater, the association between swimming and developing gastrointestinal illness, and the modelled degree of pollution in the water during the time of the competition.

## Methods

### Monitoring seawater pollution levels

Bacterial contamination of the sea around Copenhagen City is monitored by a real-time bathing water quality forecast system developed by The DHI Group in Denmark [13]. In brief, electronic systems monitor the water flow in the majority of the Copenhagen sewer networks that discharge directly or indirectly to the marine areas, and thereby predict the bathing water quality in the waters surrounding Copenhagen. This is done using a 3-dimensional hydrodynamic model describing water flows, water temperature and salinity based on wind speed and direction, air temperature, humidity and short and long wave solar radiation. The model is superimposed with a process model describing the decay of *E. coli* and enterococci based on assumptions on water transparency and inputs on solar radiation, water temperature and salinity. In the event of an observed sewer overflow and modelled concentrations of the two indicator bacteria exceeding the EU Bathing Water Directive criteria for acceptable water quality (500 *E. coli* or 200 enterococci per 100 ml), the municipal authorities are notified and will then issue a swimming ban.

### Cohort study questionnaire investigation

A total of 1,312 individuals completed the full 2010 triathlon competition (out of 1,483 participants including 171 from the relay teams). Of these 46% were Danish nationals, with the majority of the foreign nationals coming from neighbouring European countries. After the race an electronic questionnaire was distributed via e-mail to all participants in collaboration with the organizers. The questionnaire contained questions relating to symptoms, severity of disease and treatment following the competition as well as questions concerning various potential exposures such as food and beverages offered to the participants before, during and after the competition. Furthermore, participants were asked if they believed that they had swallowed water during the swimming competition and if so, approximately how many mouthfuls, and also if they thought their symptoms could have been caused simply by exhaustion. All participants were furthermore asked if they had submitted a stool sample for analysis. To assess the baseline illness caused by swimming in non-polluted seawater, a separate and comparable questionnaire investigation was conducted by e-mail after the 2011 competition. In 2011, 1,365 individuals, of whom 66% were Danish nationals, completed the full 2011 triathlon competition (out of 1,713 participants including 348 from the relay teams) which was held at the same time of year and at the same venue. 

### Data analysis

The relationship between disease and exposures was assessed using exposure-specific attack rates (AR) and relative risk of disease (RR). To examine the dose-response relationship between water intake and illness, the number of mouthfuls of ingested water was grouped into eight intervals consisting of zero, one, two, three, four to five, six to nine, and ten or more mouthfuls. The increase in odds per increasing interval was calculated using logistic regression in SAS 9.1 (SAS Institute, NC, USA). Data analysis was also performed using Stata10 (StataCorp, TX, USA).

### Ethics statement

According to Danish regulations, ethical clearance by the National Committee on Health Research Ethics is not needed for questionnaire surveys that do not involve analysis of human biological samples. The research in question did not involve analysis of human biological samples. Individual questionnaire records were only used if participants indicated that they had submitted stool samples for microbiological analyses and had accepted to be contacted directly by the Statens Serum Institut. The participant’s physician or hospital (in Denmark as well as abroad) was then consulted by phone to obtain and verify the microbiological test results. The investigation was approved by the Danish Data Protection Agency (record number 2008-54-0474).

### Case definitions

For the cohort studies, cases of gastroenteritis were defined as participants in the competitions who indicated having suffered diarrhoea or vomiting in the days following the competitions. A second, more specific, case definition was also explored consisting of participants who indicated having had diarrhoea and at least two of the following symptoms: vomiting, stomach cramps, fever, nausea, dizziness or headache.

## Results

### Modelled concentrations of bacterial pollution

**Figure 1:**
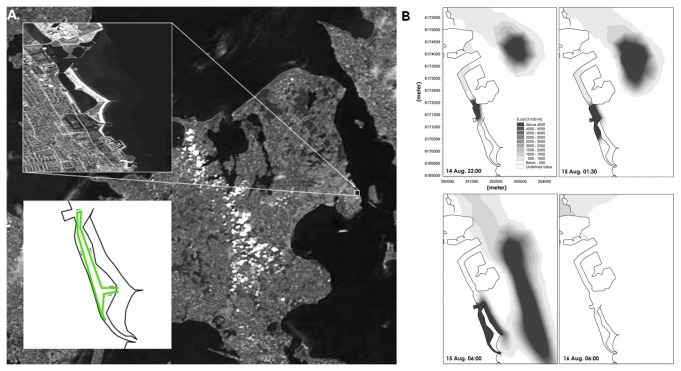
The modelled concentrations of *E. coli* in the coastal area east of Copenhagen, August 2010. Panel A shows a satellite photo of the Danish island of Zealand including the Copenhagen area, where the swim course took place in a coastal lagoon. The swim route is shown in green. Panel B shows the modelled concentrations of *E. coli* in the lagoon and surrounding area in August 2010 at: a) 10:00 p.m. 14 Aug, b) 1:30 a.m. 15 Aug, c) 6:00 a.m. 15 Aug (shortly before the competition began), and at d) 6:00 a.m. 16 Aug. CSO124 indicate the sewage overflow discharge point and WWTP the bypasses from the waste water treatment plant.

In both 2010 and 2011, the 3.8 km swimming competition followed the same route and took place in the Amager Beach Park Lagoon in the sea immediately east of Copenhagen, Figure 1. Participants were sent off in groups beginning at 7 a.m. Following the heavy rainfall in Copenhagen on the evening of 14 August 2010, data from the Copenhagen sewer monitoring system showed overflow from a number of the sewer discharge outlets. The nearest outlet close to the entrance of the lagoon (Figure 1, panel B) started discharging at 7:45 p.m. and continued until 0:15 a.m., while the bypass from the nearby waste water treatment plant continued discharging untreated bypass water until 3:45 p.m. on the 15^th^ of August. In the central part of the lagoon, threshold values of *E. coli* concentrations were exceeded at 1:30 a.m. on the 15^th^ of August 2010, and peaked between 6 and 7 a.m. in the morning (Figure 1). Modelled peak concentrations reached 2.6×10^4^ *E. coli* and 6×10^3^ enterococci per 100 ml during the morning hours and thereafter fluctuated between 1.6×10^4^ and 1.4×10^4^ *E. coli* per 100 ml. Microbial concentrations returned below threshold value again on the morning of 16 August 2010. In contrast, on the day of the 2011 competition, the modelled levels of *E. coli* and enterococci concentrations were well below the threshold of the EU Bathing Water Directive criteria (500 *E. coli* or 200 enterococci per 100 ml).

### Cases, symptoms and aetiologies

The 2010 competition questionnaire was completed by 838 participants (response rate 57%) with a median age of 36 years. A total of 351 (42%) respondents met the case definition of having suffered diarrhoea or vomiting. The 2011 questionnaire was completed by 931 participants (response rate 54%) with a median age of 38 years, of whom a total of 78 (8%) met the case definition. 

Gastrointestinal symptoms, diarrhoea, vomiting, stomach cramps and fever were significantly more pronounced among participants in the 2010 competition compared to the 2011 group (Table 1). In addition the duration of symptoms among 2010 participants that became ill lasted longer than in 2011, in particular for diarrhoea, where the median duration was 5 days in 2010 compared to 2 days in 2011 (Table 1). Furthermore, 53% of the cases in the 2011 competition, answered yes when asked if their symptoms might be due to exhaustion, compared to 13% in 2010 (Table 1).

**Table 1:** Demographic characteristics, number of cases, symptoms and indicators of severity of disease among respondents following the 2010 and 2011 triathlon competitions, Copenhagen, Denmark.

	**2010 competition (n=838**)			**2011 competition (n=931**)		
**Category**	**Number (%**)	**Median duration (days)**		**Number (%)**	**Median duration (days)**	**p-value**
**Demographics**						
Age, < 38 years	395 (47.1)	NA		545 (58.5)	NA	<0.001
Female sex	112 (13.4)	NA		126 (13.5)	NA	NS
Danish nationality	600 (71.6)	NA		750 (80.9)	NA	<0.001
**Cases**						
Cases^1^	351 (42)	NA		78 (8)	NA	<0.001
Secondary case definition, cases^2^	136 (16)	NA		34 (4)	NA	<0.001
**Individual symptoms**						
Diarrhoea	323 (39)	5		60 (6)	2	<0.001
Vomiting	115 (14)	1		27 (3)	1	<0.001
Stomach cramps	245 (29)	3		66 (7)	1	<0.001
Fever	142 (17)	2		79 (8)	1	<0.001
Nausea	207 (25)	2		139 (15)	1	<0.001
Bloody diarrhoea	9 (1)	3		5 (0.5)	2	NS
Headache	124 (15)	2		80 (9)	1	<0.001
Tenesmus	162 (19)	4		33 (4)	2	<0.001
Muscle pain	213 (25)	3		468 (50)	3	<0.001
**Severity of disease**						
Visit physician	116 (14)	NA		33 (4)	NA	<0.001
Absence from work	220 (26)	NA		27 (3)	NA	<0.001
Faeces sample submitted	48 (6)	NA		3 (0.3)	NA	<0.001
Cases reporting exhaustion	44 (13)	NA		41 (53)	NA	<0.001

p-values assessed by X^2^ test except for age where the Student t-test was used. NS = Not significant; NA = Not applicable

^1^Participants suffering from diarrhoea and/or vomiting.

^2^Participants suffering from diarrhoea and at least two of the following symptoms: vomiting, stomach cramps, fever, nausea, dizziness or headache.

Following the 2010 competition, 48 participants indicated having submitted one or more stool samples for analysis. These samples had generally been analysed for the presence of intestinal pathogenic bacteria and in a few instances also for parasitic pathogens (21%), whereas the presence of viral pathogens was rarely examined (6%). Eleven (23%) of the participants who submitted stool samples for analysis had positive analysis results, with *Campylobacter jejuni* being isolated in three samples, *Giardia lamblia* in three samples and diarrhoeagenic *E. coli* in five samples. The latter consisted of two findings of enteropathogenic *E. coli* (EPEC) and three findings of enterotoxigenic *E. coli* (ETEC). Following the 2011 competition, three participants submitted stool samples for analysis and in all three instances these contained *Campylobacter* *spp.*

### Determinants of disease

**Figure 2:**
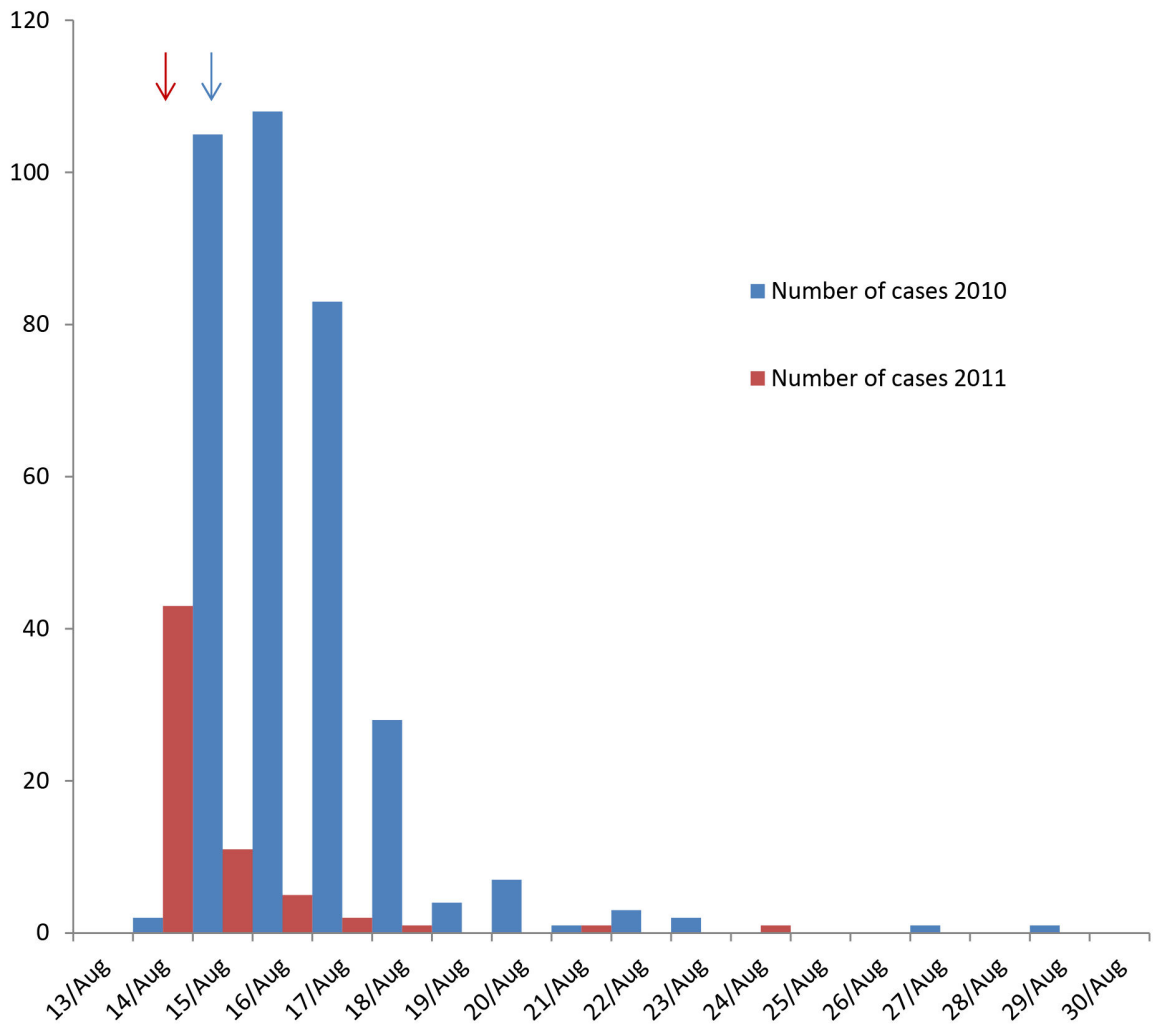
The number of cases by date of illness onset following the 2010 and 2011 triathlon competitions, Copenhagen. Day of onset of illness for cases with known onset date following the 2010 competition (blue bars, n= 345), and the 2011 competition (red bars, n=64). The 2010 competition took place on 15 August (blue arrow) and the 2011 competition on 14 August (red arrow).

A peak of reported illness was observed one day after the competition in 2010 and on the day of the competition in 2011, which was followed by a steadily declining occurrence of participants with illness in both years (Figure 2). In the 2010 competition, the only putative risk factor that was statistically significantly associated with illness was intake of seawater during swimming (Table 2). This association became stronger using the secondary case definition. The same was not observed for the other examined exposure variables. Elevated relative risks, albeit not statistically significant, were also seen for the 2011 competition (p-value for cases swallowing water = 0.07, chi-square test).

**Table 2:** Exposure specific attack rates, relative risks and 95% confidence intervals, Triathlon competitions, Copenhagen, 2010 and 2011.

		**Exposed**				**Unexposed**					
**Exposure**		**Total**	**Cases**	**AR**		**Total**	**Cases**	**AR**		**RR**	**95% CI**
**2010 competition:**											
	Participated in pre-competition dinner^1^	354	157	44.4		484	194	40.1		1.1	0.94-1.3
	Participated in social event with meals^2^	31	12	38.7		806	338	41.4		0.92	0.59-1.4
	Food 1 served during competition^3^	722	289	40.0		110	57	51.8		0.77	0.63-0.94
	Food 2 served during competition^4^	724	301	41.6		102	45	44.1		0.94	0.75-1.2
	Swallowed water^5^	475	239	50.3		172	35	20.4		2.5	1.8-3.4
	Swallowed water, secondary case definition^6^	475	105	22.1		172	9	5.2		4.2	2.2-8.2
	Female gender	112	59	52.7		726	292	40.2		1.3	1.1-1.6
	Non-Danish nationality	238	107	45.0		600	244	40.7		1.1	0.93-1.3
	Age <38 years	395	176	44.6		443	175	39.5		1.1	0.96-1.3
**2011 competition:**											
	Participated in social event with meals	319	30	9.4		612	48	7.8		1.2	0.78-1.9
	Swallowed water	540	50	9.3		211	11	5.2		1.8	0.94-3.3
	Swallowed water, secondary case definition	540	24	4.4		211	4	1.9		2.3	0.82-6.7
	Female gender	126	20	15.9		805	58	7.2		2.2	1.4-3.5
	Non-Danish nationality	177	26	14.7		750	52	6.9		2.1	1.4-3.3
	Age <38 years	545	48	8.8		386	30	7.8		1.1	0.73-1.8

AR = attack rate; RR = relative risk; CI = confidence interval.

^1^Dinnerparty arranged by the Copenhagen Challenges organizers for the participants the evening before the Ironman competition 2010.

^2^Social events arranged by the participants themselves, e.g. the different Triathlon teams.

^3^Snack at the first transition zone (T1) and during the bike course.

^4^Snack at the second transition zone (T2) and during the marathon run course.

^5^Participants suffering from diarrhoea and/or vomiting.

^6^Participants suffering from diarrhoea and at least two of the following symptoms: vomiting, stomach cramps, fever, nausea, dizziness or headache.

Respondents from the 2010 study who indicated having swallowed water during the swim course were further inquired about the number of ‘mouthfuls’ of water that they believed they had swallowed. The median number of mouthfuls swallowed was 3 (range 1-200). After grouping the number of mouthfuls into intervals there was a moderate, but statistically significant, relationship between illness and the dose of seawater ingested. When calculated using logistic regression, the odds ratio (OR) rose with 1.19 (95% confidence interval (CI): 1.10-1.28) per interval. Using the secondary, more stringent, case definition, the OR was found to rise with 1.23 (95% CI: 1.11-1.35) per increase in interval.

**Figure 3:**
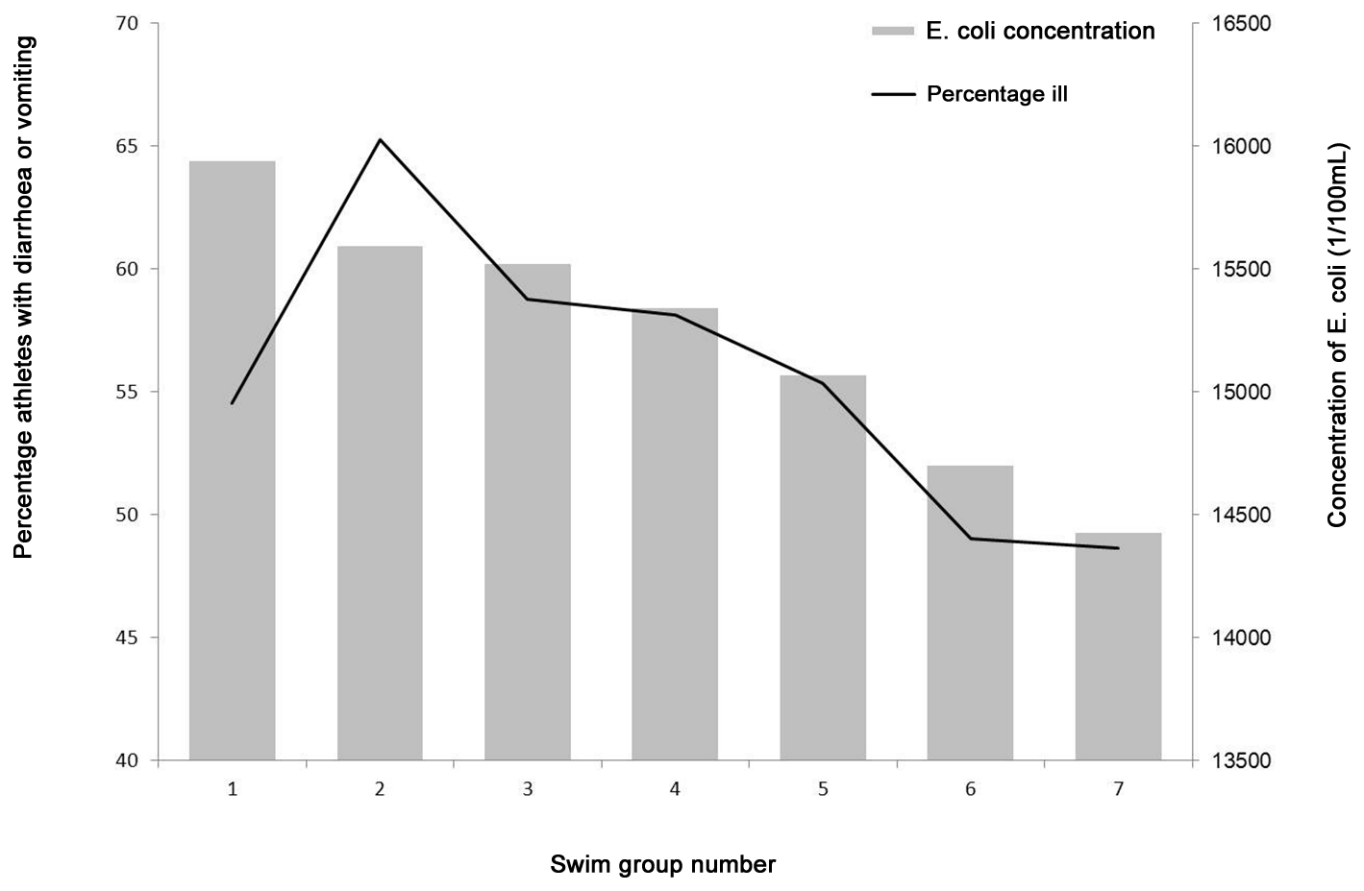
The self-reported illness and the modelled *E. Coli* concentrations during the 3.8 km open water swimming competition in 2010, Copenhagen. Percentage of self-reported illness (line) in each of the seven swim groups of the 2010 competition and modelled *E. coli* concentrations during the competition (bars). Group 1 consisted of professionals (mean swimming time 55 minutes), group 2 of women and seniors (mean swimming time 81 minutes) and groups 3-7 of men at different ages (mean swimming time between 65-79 minutes), grouped by starting number.

Investigating two cohorts of swimmers enabled a comparison between the disease risk in the two years as well as other characteristics of the swimmers. The attack rates of swimming in polluted and unpolluted water were 42% versus 8%, giving a relative risk (RR) of 5.0 (95% CI: 4.0-6.3) of developing gastroenteritis when exposed to the polluted water. In both competitions, relatively more women than men were affected, with the RR of having female gender being 1.3 (95% CI: 1.1-1.6) in the polluted water (2010 competition) and 2.2 (95% CI: 1.4-3.5) in the unpolluted. Swim time and swim group were not associated with illness in either competition, however the rate of self-reported illness in the different swim groups (disregarding swim group 1, which consisted of professional athletes, n=17, who spent considerably less time in the water than the following amateur groups) followed the concentration of *E. coli* to which the swimmers were exposed, as shown in Figure 3. Nationality was not associated with illness in the 2010 competition (RR 1.0, 95% CI: 0.9-1.3), but in 2011 non-Danish athletes had a higher risk of illness (RR 2.1, 95% CI: 1.4-3.3) (Table 2).

## Discussion

In this study, we used a rare natural event to demonstrate the health effects associated with recreational contact to sewage-polluted coastal water compared to non-polluted water. We observed a large group of physically fit individuals swimming in water contaminated with what corresponded to an average of 1.5x10^4^ *E. coli* bacteria per 100 ml. For this group of people, gastrointestinal illness occurred five times more often than in a group of athletes swimming the same distance in the same area under non-polluted conditions. Furthermore, symptoms in the first group were of significantly longer duration. Unintentionally swallowing seawater whilst in the sea was a significant risk factor for disease, with an increased risk of illness in people who ingested higher doses of water. 

The association between recreational swimming in coastal waters and developing a range of symptoms and illnesses has previously been demonstrated [8,14,15], and this study further confirms such a relationship. The 2010 Copenhagen Ironman triathlon competition was held the day after an unusually heavy rain fell on the greater Copenhagen area. Following the rainfall, a bathing water quality forecast model indicated a substantial sewage pollution of the coastal waters in which the swimming competition took place. Heavy rainfall overloaded the sewage systems and led to sewage overflowing into surrounding urban and aquatic areas. The bathing water quality forecast model has been implemented by the City of Copenhagen as an early warning system which is run on a daily basis. Data from the model used in this study showed that the bacterial contamination in the sea peaked during the morning of the swimming competition, causing a significant outbreak of gastrointestinal disease among the participants. The evidence linking disease to the sewage-polluted swimming water is further strengthened by the fact that different bacterial and parasitic pathogens were isolated from stool samples. However, as also indicated by the widely varying duration of symptoms, illness was most likely caused by a broader register of different agents. In most European laboratories, patient stool samples are generally only analyzed for bacterial pathogens and rarely for parasites or viruses, which to some degree may explain the high prevalence of negative laboratory results among the exposed swimmers in our study. Due to the mixed etiology and diverse range of symptoms we chose a sensitive case definition in this study, although also exploring a second more strict case definition. As expected for a causal relationship, a stronger association between illness and ingestion of water was seen when the latter was applied.

In 2011, although the bathing water quality forecast model indicated that the coastal waters were not contaminated during the day of the competition, a number of symptoms were still reported. Compared to 42% in 2010, only 8% of the 2011 athletes were classified as cases, with symptoms generally of much shorter duration. In addition, more cases in the 2011 study indicated that their symptoms were likely due to exhaustion. Although not statistically significant, the athletes from the 2011 cohort who unintentionally ingested seawater had an elevated risk of illness, and stool samples from three athletes were confirmed positive for the presence of *Campylobacter*. This indicates that there is a modest but real risk of developing gastroenteritis after prolonged swimming even when pollution levels are within the accepted interval. This is supported by the findings of a study of triathletes in the Netherlands [16] which demonstrated an increased risk of gastroenteritis among athletes who participated in the swimming course compared to those who did not swim, in spite of the fact that the water quality met the current EU Bathing Water Directive.

The extreme rainfall preceding the 2010 Copenhagen Ironman competition caused significant flooding in most of Copenhagen. In Denmark, such heavy rainfalls have recently occurred more frequently during the summer months when people spend more time outside, often in contact with recreational water and therefore also increasingly likely to be exposed to either sewage contaminated seawater or flood waters in streets, parks etc. Indeed, climate changes are predicted to have a significant impact on rainfall in the Northern Hemisphere, causing more frequent intense precipitation events [17] and a higher likelihood of sewage overflow and contamination of recreational water facilities [18-20]. In cities such as Copenhagen where sewage systems are not designed to accommodate sudden major increases in water flow, the potential economical and health-related impacts could be substantial. This was illustrated in July 2011 when another extreme flooding episode occurred in Copenhagen, causing several confirmed cases of, and one death from, leptospirosis amongst persons who emptied their basements from sewage-polluted flood water [21,22]. Combined with this, the findings from our study of swimming athletes can be used as a model example to highlight the risk of disease in future flooding episodes. Further quantifying the relationship between patchy contact with sewage-contaminated water and the risk of disease using water quality modelling will not only allow the identification of high-risk behaviours and populations and areas at risk but also be an important tool for future water management and disaster planning in vulnerable locations.

Several limitations may have affected this study. In 2010 the questionnaire was launched one month after the competition, whereas in 2011 the questionnaire was launched after two weeks, which could give a possibility for differential recall. In addition, the participants in the 2010 competition were exposed to media stories of athletes that had suffered from diarrhoea and abdominal pain after the competition, which might have led the 2010 participants to remember any gastrointestinal symptoms more clearly. Furthermore, the lack of a systematic examination of stools from participants naturally limits the conclusions as to which disease agents may have caused the observed symptoms.

In conclusion, this study among two cohorts of athletes demonstrates a significant risk of illness associated with contact with sewage-polluted recreational water that exceeds the threshold of 500 *E. coli* or 200 enterococci per 100 ml. The study adds another dimension to previously published studies of water-borne disease outbreaks by showing that after an extreme rainfall even physical contact with and unintentional ingestion of small amounts of sewage-polluted flood water can lead to pronounced gastrointestinal illness. In a climate with a predicted increasing frequency of heavy rainfall, the future implications for human health on a world-wide basis could be considerable, particularly in those areas without well-functioning sewage systems or where current sewage systems are unable to contain the excess masses of water.
